# Impacts of workplace automation on energy poverty: The new challenge of achieving SDG 7 in the context of technological revolution

**DOI:** 10.1016/j.heliyon.2024.e25087

**Published:** 2024-01-23

**Authors:** Xiaoru Niu, Chao Li, Xiang Li, Yuhan Zhang

**Affiliations:** a School of Mechanical, Electrical & Information Engineering, Shandong University, 180 Wenhuaxi Road, Weihai, 264209, China; b Business School, Shandong University, 180 Wenhuaxi Road, Weihai, 264209, China; c Centre for Quality of Life and Public Policy Research, Shandong University, 72 Binhai Road, Jimo, Qingdao, 266237, China; d HSBC Business School, Peking University, University Town, Shenzhen, 518055, China

**Keywords:** Workplace automation, Energy poverty, Sustainable development goal 7, Technological revolution, Energy consumption

## Abstract

This paper systematically examines how workplace automation impacts energy poverty from a demand-side perspective, revealing a new challenge for Sustainable Development Goal 7 (SDG 7) in the context of technological revolution. Our research demonstrates that workplace automation significantly increases household energy poverty. This finding is robust when using the instrumental variable approach to tackle endogeneity, as well as employing different automation and energy poverty measures, placebo tests, and machine learning methods for robustness checks. Automation's impact mechanism is that it reduces people's income and work-related social capital, thus exposing households to higher risks of energy poverty. Moreover, its consequences are more prominent for rural households, less educated people, non-migrants, those without labor contracts, non trade-union members, and out-of-system workers. Thus enhancing human capital, promoting free movement of workers, and providing better labor protection contribute to weakening the adverse impact of the technological shock. Meanwhile, we find that improving the price reasonability, stability, security and accessibility of energy supply can also mitigate the negative effects of workplace automation on household energy consumption. In the dual context of the fourth technological revolution promoting industrial automation as well as the increasing urgency to achieve SDG 7, findings of this paper have important policy implications.

## 1 Introduction

Energy poverty is one of the major challenges in the global energy system today [1]. Currently, 733 million people worldwide still live without electricity and 2.4 billion people lack access to clean fuels and modern technologies for cooking [2]. Energy poverty has significant detrimental effects on economic development [3], productivity [4], social well-being [5], as well as people's quality of life [6]. For these reasons, to ensure access to affordable, reliable, sustainable and modern energy is listed as the seventh goal of United Nations' Sustainable Development Goals, abbreviated as SDG 7. However, the latest *2022 Tracking SDG 7: The Energy Progress Report*, collectively issued by International Energy Agency (IEA), International Renewable Energy Agency (IRENA), United Nations Statistics Division (UNSD), World Bank and World Health Organization (WHO), declares that at today's rate of progress, the world is still not on track to achieve the SDG 7 by 2030.

In this context, many studies have delved into the potential influences of energy poverty from various aspects such as income, education, and industrial structure. Indeed, as a new engine driving the rapid development of the global economy, the impact of automation is worth exploring, because innovative technologies have created more opportunities and forces to expand the global energy system. According to the IEA's report, investment in the automation and digitalization of energy systems has experienced a significant increase. This surge is attributed to new technologies that possess the capability to enhance energy supply, facilitate demand response, and predict faults. For example, global investment in digital electricity infrastructure and software has grown by over 20 % annually since 2014, reaching USD 47 billion in 2016. The above analysis from an energy supply perspective seems to suggest that automation improves the energy supply efficiency, thereby contributing to eliminating energy poverty. However, the effects of workplace automation on household energy affordability and accessibility from the energy demand perspective remain unexplored. Therefore, this study aims to investigate a new challenge facing SDG 7 in the context of the ongoing technological revolution.

Given China's notable achievement as one of the countries experiencing the swiftest industrial automation and exerting significant global influence over the past decade, this study centers its focus on China. Presently, China boasts the world's largest robotics market. In 2022, the number of newly installed robots in China reached 290258, accounting for 52.482 % of the world's total new installations. China's remarkable automation achievements create a favorable opportunity to examine its impact on household energy poverty. In addition, according to the IEA, China's final energy consumption in 2022 is 106.912 (EJ), representing 24.167 % of total global energy consumption. China has been the largest energy consumer, but its energy consumption in the residential sector accounts for only 16.758 % of the country's total final energy consumption. This percentage is significantly lower than the world average of 21.001 %. Consequently, a question arises: does automation indeed alleviate energy poverty?

In the dual contexts of the fourth technological revolution spearheaded by AI promoting industrial automation as well as the increasing urgency to achieve SDG 7, it is particularly necessary to clarify the impact of automation on energy poverty. In light of this, this paper empirically investigates the effect of workplace automation on energy poverty using the Chinese General Social Survey (CGSS). Besides, endogeneity and robustness checks from multiple aspects are conducted. Furthermore, we also explore the mechanisms of economic income and work-related social capital through which automation affects energy poverty. In addition, this research analyzes heterogeneities of automation's consequences and possible pathways to address the challenge from the perspective of demographic characteristics, labor protection and energy supply.

The contributions of this paper are reflected in the following two aspects. First, this paper identifies a new challenge facing SDG 7 from a novel technological disruption perspective. There are many factors influencing energy poverty. Previous literature has mainly focused on economic income, family demographic and social characteristics, as well as regional factors, but has relatively neglected the impact of the new technological change on energy poverty. This factor is gaining importance under the background of the current fourth technological revolution. Therefore, this study helps to explain the problem of energy poverty and the challenges threatening progress towards Sustainable Development Goal 7 from a new perspective. Second, this paper examines the impact of digital disruption on energy markets from a demand perspective. Previous studies have primarily focused on the supply-side effects of automation on energy markets. Studies suggest that the positive influence of automation on energy efficiency and energy structure is beneficial to addressing the challenge of energy poverty. For instance, automation technologies represented by artificial intelligence could optimize the energy supply through intelligent algorithms and automated devices, thereby diminishing waste and enhancing energy supply efficiency. Besides, the application of automation in various industries reduces the demand for traditional energy sources and facilitates the energy transition. However, to the best of our knowledge, the indirect impact of automation on energy poverty, which is a deep-rooted socioeconomic factor, has not been investigated in preceding studies. This paper provides a valuable exploration in this regard.

The remainder of the paper is organized as follows: Section 2 is literature review and theoretical background. Section 3 presents the data and methodology. Section 4 contains the results of the benchmark analysis, endogeneity checks and robustness tests. Section 5 examines the impact of workplace automation on different energy poverty measures. Section 6 analyzes the impact mechanisms of automation. Section 7 conducts heterogeneity analysis. Section 8 discusses the results. Section 9 summarizes findings of this paper and puts forward policy implications.

## 2 Literature review and theoretical background

### 2.1 Effects of workplace automation

Automation technology and its widespread applications have been profoundly impacting many sectors in multiple ways. For the energy resources market, established studies have mostly examined the favorable effects of automation on improving energy supply efficiency and promoting energy transition from the supply side [7,8]; . Consequently, the advancement of automation technology stands out as a significant factor in solving the problem of energy poverty. Specifically, automated processes help to streamline operations, minimize energy wastage and optimize resource utilization, ultimately enhancing energy supply [[7], [8], [9]]. For example, some research finds that automation technology significantly contributes to economic growth and energy supply efficiency in South Asian economies [10]. However, others reveal the opposite conclusion, stating that automated equipment is not energy efficient and, in fact, lowers energy efficiency [11]. Additionally, integrating automation technology into various industries reduces their reliance on traditional energy sources, facilitating industrial upgrading and promoting energy transition. Research indicates that automation technology effectively drives the transition energy production towards renewable models, and this impact is observed in 72 countries globally [9]. Despite some controversies, most studies emphasize the benefits of automation on energy supply efficiency and energy structure transformation from a supply perspective, which contributes to reducing energy poverty.

In addition to its role in the energy market, the impact of automation on the labor market should not be ignored. Most studies confirm its positive effects on economic development, productivity, and enterprise innovation [12,13]. In addition to bringing huge benefits to the economy, researchers who are optimistic about automation also consider it to be “labor friendly” owing to the following reasons. First, automation has productivity effect. It is found that automation applications not only help to increase the total output but also improve the quality of economic growth [14]. Important reasons for this are that automation promotes productivity and thus increases workers' income and employment [[15], [16], [17]]. As employment opportunities rise, workers' social capital accumulated in the workplace also increases. This is because work not only enhances social capital through facilitating interpersonal interactions, building trust, and sharing experiences, but also expands workers' social connections [[18], [19], [20]]. Second, automation has complementarity effect on labor demand. When automation is applied to production, it requires close cooperation of workers’ cognitive and interpersonal skills, raising employment and earnings of workers with such skills [21,22]. This also leads workers to seek further learning and training to acquire these skills to achieve human-machine collaboration, thereby increasing their wages [23]. Third, automation brings cost-saving effect. The application of automation can save production costs, lower the price of products and increase product demand, for which labor is in greater demand and labor income can be lifted [24]. Furthermore, the expanding demand for labor makes workers more likely to enter the labor market and participate in social interactions, thus promoting the accumulation of their social capital [20]. Because of these facts, automation may exert positive effects on employment and income.

However, another stream of literature is pessimistic about automation, arguing that it has negative impacts on employment, income, and work-related social capital. First of all, in terms of employment and income, automation technology has the effect of replacing workers. This is due to its capital deepening and labor-saving impacts, which cause people's jobs to be substituted and income to decline [[25], [26], [27]]. Moreover, workers who experience job loss are not only directly impacted economically but also face a reduction in their social capital, stemming from their inability to engage in resource-sharing and social interactions in the workplace [28]. In addition, automation also has a mismatch effect on the labor market. Due to the long time needed for human capital investment, workers' skills may not meet the requirements of the new technology in the short term. As a result, the economic benefits brought by automation are reduced since there is a mismatch between labor skills and the demand for workers by new technologies [29]. Therefore, the automation-induced mismatch creates more risk of frictional unemployment and reduces labor income [[30], [31], [32]]. Research indicates that unemployment leads to social withdrawal among workers, impacting their sustained involvement in social networks and, consequently, proving detrimental to the accumulation of social capital [28].

### 2.2 Energy poverty and its possible relationship with workplace automation

Energy poverty is one of the three major challenges in the world energy system [1,33], and has received widespread attention from both the academia and policymakers. There are many factors influencing energy poverty, the most fundamental of which is the economic income. On the one hand, income determines the energy choice of households. According to the energy ladder theory [34], as income increases household energy consumption patterns evolve through a ladder from traditional biomass to modern clean fuel. Nevertheless, higher income levels may lead to increased biomass and non-clean energy consumption among impoverished households [35]. This phenomenon may be attributed to the inadequate development of energy infrastructure in economically disadvantaged areas, posing challenges for households to access clean energy [36]. In addition to economic income, family demographic and social characteristics can also exert influences on energy poverty. Studies have shown that the more children, the greater the burden of child-rearing, and the higher the likelihood of energy poverty [37]. However, larger family sizes may improve energy efficiency through the energy-sharing effect [38]. In terms of marital status, owing to the risk-sharing effect of marriage, it can reduce energy poverty [39]. Ethnic minority households in many developing countries still rely on coal and traditional biomass for their energy needs [40,41]. As a result, they are more likely to face energy poverty problem. This issue also prevails in developed countries, as evidenced by higher rates of energy poverty among African American households compared to White and Asian ones [42]. Additionally, household head's age [43,44], gender [45,46], and education [1,46,47] are also important factors affecting energy poverty. For example, higher education level usually means higher awareness and more knowledge of energy saving and environmental protection and thus the improvement of education level help to increase the consumption of clean energy while reducing the traditional biomass energy consumption. Therefore, enhancing education contributes to the transformation of household energy consumption, thus helping alleviate energy poverty [48,49]. Besides intra-household factors, regional elements such as industrial structure [50], marketization [51], financial system [52], and green fiscal policy [53] also explain variations in energy poverty across regions.

The relationship between workplace automation and energy poverty has not yet been directly discussed in previous literature. From theoretical analysis, we can indirectly speculate the relationship between the two. On the one hand, due to its productivity effect [13], complementarity effect [21,22], and cost-saving effect [24] discussed above, automation may promote employment and workers’ income and social capital [15,54,55]. Meanwhile, high-income households are less likely to fall into energy poverty, since the proportion of their energy expense in total expenditure is relatively lower and their energy consumption is cleaner and more modern [[56], [57], [58]]. Additionally, higher social capital contributes to mitigating household energy poverty [1,59]. Based on this, Hypothesis 1 can be proposed: workplace automation decreases energy poverty.

However, on the other hand, automation technology may replace workers and reduce their income and work-related social capital [[25], [26], [27]]. Besides, for the labor market, automation can also lead to a mismatch between labor skills and the demand for workers by new technologies, resulting in frictional unemployment and lower income, as well as decreased social capital [[30], [31], [32]]. Also, households with lower income tend to use more low-quality solid fuels and spend a larger share of their total income on energy [35,36], which makes them more vulnerable to energy poverty. Furthermore, a decline in social capital reduces the ability to convert it into physical capital, increasing the likelihood of energy poverty and exacerbating overall poverty [59]. Therefore, in view of this, Hypothesis 2 can be proposed, which is the opposite of Hypothesis 1: workplace automation increases energy poverty.

**Fig. 1:**
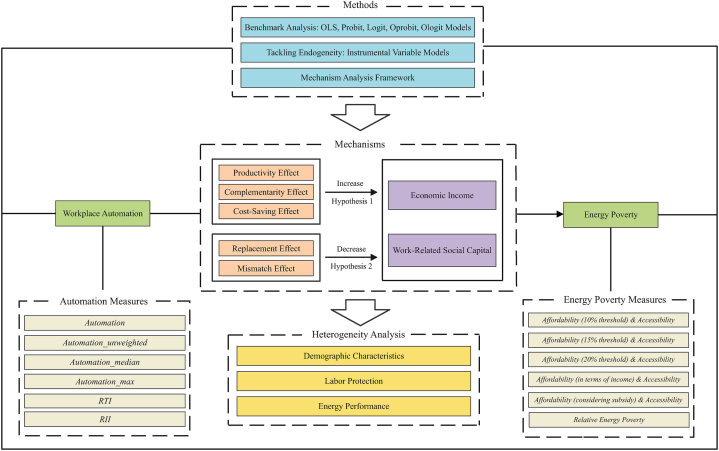
Research framework.

In summary, studies on consequences of automation have mainly focused on its direct effects on output, productivity, employment and income, while the indirect impact of automation on energy poverty which is an important socioeconomic factor has not been emphasized. Although existing literature has accumulated valuable results on factors influencing energy poverty, there is still a research gap with regard to analyzing causes of energy poverty from the new perspective of technological progress. Moreover, from theoretical analysis, the impact of automation on energy poverty is still unclear. In view of the above, using the Chinese General Social Survey (CGSS), this paper systematically examines the impact of workplace automation on energy poverty. The research framework is shown in Fig. 1.

## 3 Data and methodology

### 3.1 Data sources

The data used in this paper are mainly from Chinese General Social Survey (CGSS), one of the most important national, comprehensive, and continuous academic survey projects in China. It aims to systematically and comprehensively collect multifaceted information on Chinese society and track trends in social change. Its sample covers 28 provinces/municipalities/autonomous regions in China, which is highly representative of the country. Besides, CGSS has two advantages for this study: (1) CGSS investigates household total expenditure, income, and detailed information on energy consumption in aspects of electricity, honeycomb coal, briquettes, coal lumps, liquefied gas, pipeline gas, livestock manure, straw, firewood, etc. This enables us to construct energy poverty indicators integrating affordability and accessibility, and test the robustness of automation's effect on energy poverty from multiple perspectives. (2) CGSS contains detailed family representatives' occupational codes of International Standard Classification of Occupations 2008 (ISCO2008), facilitating measuring the impacts of workplace automation on them. CGSS currently has 12 waves of data from 2003 to 2018. Since the occupational codes in the data before 2017 are not ISCO2008 standard and household energy use information is not investigated in 2017, this paper uses the 2018 wave data in CGSS.

### 3.2 Dependent variables

**Fig. 2:**
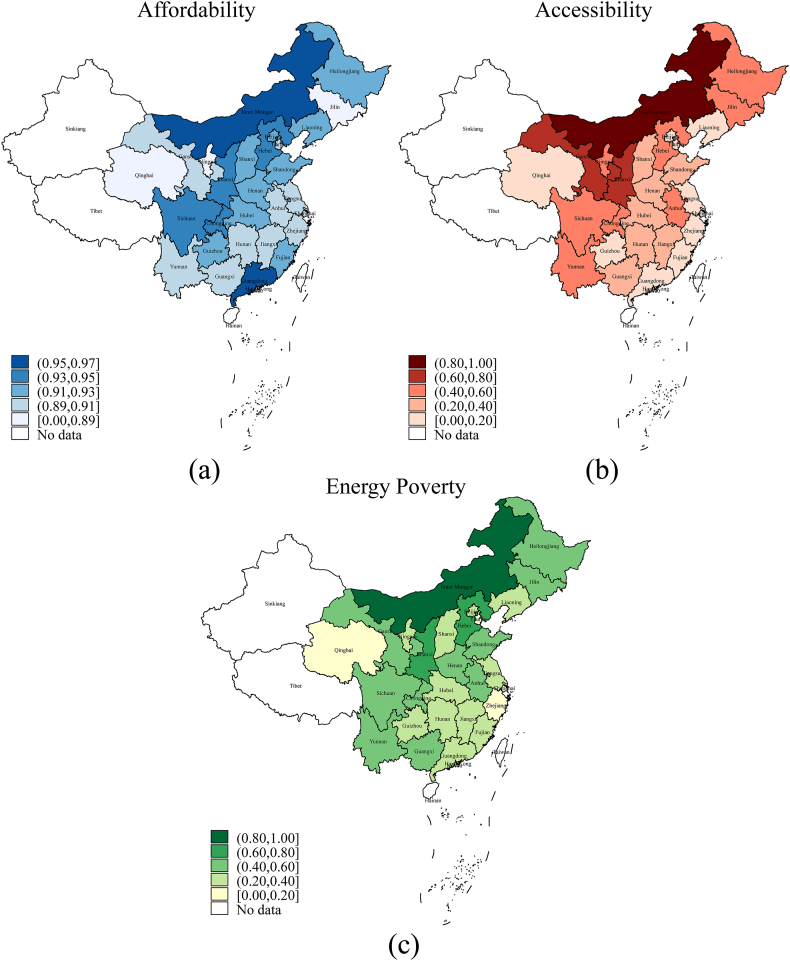
(a) Spatial distributions of the affordability measure of energy poverty, (b) spatial distributions of the accessibility measure of energy poverty, (c) spatial distributions of the composite energy poverty measure incorporating affordability and accessibility.

The main dependent variable in this paper is energy poverty at the household level, denoted as ${Energy\_poverty}_{i}$. Referring to Zhang et al. [60], Xiao et al. [61], Du et al. [1], and Wang et al. [62], this paper constructs an energy poverty indicator combining two aspects of energy affordability and accessibility, as expressed in equation (1). Specifically, energy affordability is measured by a 10 % economic threshold. If energy expense accounts for more than 10 % of total household expenditure, the dummy variable ${Affordability\_poverty}_{i}$ in equation (2) is equal to 1, otherwise 0 [[63], [64], [65], [66], [67]]. Energy accessibility is defined by whether the household uses solid fuels. The dummy variable ${Accessibility\_poverty}_{i}$ in equation (3) is equal to 1 if the household uses low-quality solid fuels such as honeycomb coal, briquettes, coal lumps, livestock manure, straw and firewood, and 0 otherwise [68]. On this basis, integrating energy affordability with accessibility, ${Energy\_poverty}_{i}$ is constructed as equation (1). Spatial distributions of ${Affordability\_poverty}_{i}$, ${Accessibility\_poverty}_{i}$ and ${Energy\_poverty}_{i}$ are illustrated in subfigures (a)–(c) of Fig. 2, demonstrating high correlations among these indicators. Additionally, other different types of energy poverty indexes are also developed for robustness checks.

(1) $${Energy\_poverty}_{i}={0.5*Affordability\_poverty}_{i}+{0.5*Accessibility\_poverty}_{i}$$

(2) $${Affordability\_poverty}_{i}=\left\{ \begin{array}{c} 1\;if\;energy\;expenses/total\;expenses>10\%\\ 0\;if\;energy\;expenses/total\;expenses\leq 10\% \end{array}\right.$$

(3) $${Accessibility\_poverty}_{i}=\left\{ \begin{array}{c} 1\;if\;using\;low-quality\;solid\;fuels\\ 0\;if\;not\;using\;low-quality\;solid\;fuels \end{array}\right.$$

### 3.3 Explanatory variables

The main explanatory variable in this paper is the impact of automation on representative's work at the household level, constructed by Frey and Osborne [30], denoted as ${Automation}_{i}$. This indicator, based on the multidimensional characteristics of different occupations in Occupational Information Network (O*NET) of the US Department of Labor in terms of perception and manipulation tasks, creative intelligence tasks, and social intelligence tasks, uses machine learning algorithms to measure the degree of automation's impact on different occupations. It has been widely used to characterize automation's effects on people [69,70]. It is worth to mention that, this indicator is calculated based on the 2010 version of SOC (Standard Occupational Classification) occupational classification standard of the US Department of Labor, and we use the occupational crosswalks from the US Bureau of Labor Statistics to convert this index into the ISCO2008 standard measure. In this conversion, a certain ISCO2008 occupation may correspond to multiple SOC2010 occupations, for which we construct the automation indicator for ISCO2008 vocations with the employment scale of different SOC2010 occupations in the 2017 US Occupational Employment Survey (OES) as the weight. In addition, in robustness tests, this paper also employs indicators from other transformations and scholars as explanatory variables for analysis.

### 3.4 Control variables

Referring to literature on energy poverty (e.g. [45,46,50]), to avoid omitted variable bias, this paper fully controls influencing factors in the following five aspects: (1) Household income and assets characteristics include the logarithm of household income (RMB), number of housing assets and whether owning cars. (2) Family social and demographic characteristics include social status, family size, number of children, whether Hukou is in urban areas, whether being ethnic minorities and whether having religious beliefs. (3) Social security characteristics include whether having medical insurance and pension. (4) Family representative's characteristics include her/his education level, health condition, whether being migrant, age, squared terms of age, gender, whether married, whether the Communist Party of China (CPC) member, and whether working in-system. (5) Regional variations are characterized by provincial dummies. Table 1 shows the descriptions and statistical characteristics of above variables.

**Table 1:** Descriptive statistics.

Variable	Description	Obs.	Mean	Std. Dev.	Min.	Max.
Dependent Variable						
Energy_poverty	Whether in energy poverty, Yes = 1, No = 0	1988	0.403	0.352	0	1
Explanatory Variables						
Automation	Automation (weighted index)	1988	0.661	0.314	0.005	0.990
Automation_unweighted	Automation (unweighted index)	1988	0.647	0.303	0.008	0.990
Automation_median	Automation (median index)	1988	0.650	0.311	0.008	0.990
Automation_max	Automation (max index)	1988	0.713	0.309	0.008	0.990
RTI	Automation (RTI index)	1988	2.061	0.656	0	3
RII	Automation (RII index)	1983	−0.379	0.551	−1	1
Control Variables						
ln_Income	Logarithm of household income (RMB)	1818	10.537	1.673	0	14.286
Number of houses	Number of houses in the household	1971	1.092	0.643	0	11
Whether owning cars	Yes = 1, No = 0	1984	0.316	0.465	0	1
Social status	1-10 levels	1965	4.223	1.658	1	10
Family size	Number of family members	1987	2.955	1.432	1	11
Number of children	Number of children	1985	1.555	1.180	0	10
Whether Hukou in urban areas	Yes = 1, No = 0	1984	0.296	0.457	0	1
Whether ethnic minorities	Yes = 1, No = 0	1988	0.085	0.279	0	1
Whether religious believers	Yes = 1, No = 0	1988	0.092	0.289	0	1
Whether having pension	Yes = 1, No = 0	1987	0.741	0.438	0	1
Whether having medical insurance	Yes = 1, No = 0	1988	0.935	0.246	0	1
Education level	1-13 levels	1988	5.204	3.325	1	13
Health condition	1-5 levels	1986	3.699	1.035	1	5
Whether migrants	Yes = 1, No = 0	1982	0.143	0.350	0	1
Age	Age	1988	45.720	13.100	19	75
Age_squared	Squared term of age	1988	2261.883	1224.254	361	5625
Whether female	Yes = 1, No = 0	1988	0.470	0.499	0	1
Whether married	Yes = 1, No = 0	1988	0.807	0.395	0	1
Whether CPC member	Yes = 1, No = 0	1987	0.090	0.286	0	1
Whether working in-system	Yes = 1, No = 0	1977	0.091	0.288	0	1
Province dummies						

Notes: Social status is self-rated and classified from 1 to 10, with 1 denoting the lowest social status and 10 denoting the highest social status. Hukou is a system of household registration used in mainland China, mainly identifying a person as a rural or urban resident. Education level is classified from 1 to 13: 1-without any education, 2-kindergarten, 3-primary school, 4-junior high school, 5-vocational high school, 6-ordinary high school, 7-techinical secondary school, 8-technical high school, 9-junior college (adult education), 10-junior college (regular education), 11-undergraduate (adult education), 12-undergraduate (regular education), 13-postgraduate and above. Health status is based on the self-rated health levels from 1 to 5: 1-very unhealthy, 2-relatively unhealthy, 3-medium, 4-relatively healthy, 5-very healthy. Working in-system in China refers to having jobs in Communist Party of China organizations and governments.

### 3.5 Empirical models

First, the following Ordinary Least Square (OLS) model is constructed to examine the relationship between workplace automation and energy poverty as shown in equation (4).

(4) $${Energy\_poverty}_{i}=\alpha_{0}^{1}+\alpha_{1}^{1}{Automation}_{i}+x_{i}^{\prime }\psi^{1}+\varepsilon_{i}^{1}$$

In model (4), ${Energy\_poverty}_{i}$ is the energy poverty indicator. ${Automation}_{i}$ represents the degree to which family representative's work is affected by automation. $x_{i}^{\prime }$ denotes a vector of control variables described above. $\varepsilon_{i}^{1}$ is the random error term. $\alpha_{1}^{1}$、 $\psi^{1}$ refers to the parameters to be estimated.

In addition, since the values of the energy poverty indicator are 0, 0.5 and 1, lying in the range of [0, 1]. However, the predicted values obtained by the OLS model may be outside this interval. Therefore, for such explained variables with values in the interval of [0,1], the Probit and Logit models based on maximum likelihood estimation are also used to investigate the relationship between automation and energy poverty. The log-likelihood function for Probit or Logit model is of the form in equations (5), (6), (7).

(5) $$lnL=\sum_{i\in S}\ln\left[F\left(\alpha_{0}^{2}+\alpha_{1}^{2}{Automation}_{i}+x_{i}^{\prime }\psi^{2}\right)\right]+\sum_{i\notin S}\ln\left[1-F\left(\alpha_{0}^{2}+\alpha_{1}^{2}{Automation}_{i}+x_{i}^{\prime }\psi^{2}\right)\right]$$

(6) $$F\left(\cdot \right)=\Phi \left(\alpha_{0}^{2}+\alpha_{1}^{2}{Automation}_{i}+x_{i}^{\prime }\psi^{2}\right)$$

or

(7) $$F\left(\cdot \right)=\exp\left(\alpha_{0}^{2}+\alpha_{1}^{2}{Automation}_{i}+x_{i}^{\prime }\psi^{2}\right)/\left\{1+\exp\;\left(\alpha_{0}^{2}+\alpha_{1}^{2}{Automation}_{i}+x_{i}^{\prime }\psi^{2}\right)\right\}$$

In equation (5), S is the set of all observations such that ${Energy\_poverty}_{i}\neq 0$. F(·) can take two forms. One approach is to use equation (6), where Φ(·) is the standard normal cumulative density function, performing Probit estimation. The other way is to use equation (7) and perform Logit estimation. Based on this, $\alpha_{1}^{2}$ and $\psi^{2}$ are estimated by $\max_{\alpha_{1}^{2},\psi^{2}\;}lnL$.

Further, since ${Energy\_poverty}_{i}$ takes the value of 0, 0.5 or 1, which is an ordered dependent variable. We also employ the Ordered Probit and Ordered Logit models for estimation. Specifically, based on ${Energy\_poverty}_{i}$, the sample is divided into three different groups. Groups g = 1 to 3 represent ${Energy\_poverty}_{i}=0,{Energy\_poverty}_{i}=0.5\;\text{and}\;{Energy\_poverty}_{i}=1$, respectively. The probability $p_{gi}$ of a given observation i can be expressed as equations (8), (9) or equation (10).

(8) $$p_{gi}=\mathrm{Pr}\left({Energy\_poverty}_{i}=\frac{g-1}{2}\right)=\mathrm{Pr}\left(\chi_{g-1}<\alpha_{0}^{3}+\alpha_{1}^{3}{Automation}_{i}+x_{i}^{\prime }\psi^{3}+\varepsilon_{i}^{2}\leq \chi_{g}\right)$$

(9) $$\mathrm{Pr}\left(\cdot \right)=\Phi \left(\chi_{g}-\alpha_{0}^{3}-\alpha_{1}^{3}{Automation}_{i}-x_{i}^{\prime }\psi^{3}\right)-\Phi \left(\chi_{g-1}-\alpha_{0}^{3}-\alpha_{1}^{3}{Automation}_{i}-x_{i}^{\prime }\psi^{3}\right)$$

or

(10) $$\mathrm{Pr}\left(\cdot \right)=1/\{1+\exp\;\left(\alpha_{0}^{3}+\alpha_{1}^{3}{Automation}_{i}+x_{i}^{\prime }\psi^{3}-\chi_{g}\right)\}-1/\{1+\exp\;\left(\alpha_{0}^{3}+\alpha_{1}^{3}{Automation}_{i}+x_{i}^{\prime }\psi^{3}-\chi_{g-1}\right)\}$$

In expression (8), $\chi_{0}$ is −∞ and $\chi_{3}$ is +∞. Pr(·) can take two forms. One method is to use equation (9) adopting the Ordered Probit model, where Φ(·) is the standard normal cumulative distribution function. Alternatively, equation (10), the Ordered Logit model, can also be used for estimation. Accordingly, the log-likelihood of the maximum likelihood estimation (MLE) is shown as equation (11).

(11) $$lnL=\sum_{i=1}^{N}\sum_{g=1}^{3}I_{g}\left({Energy\_poverty}_{i}\right)\ln\;p_{gi}$$

where $I_{g}\left({Energy\_poverty}_{i}\right)=\left\{ \begin{array}{c} 1\;if\;{Energy\_poverty}_{i}=\frac{g-1}{2}\\ 0\;if\;{Energy\_poverty}_{i}\neq \frac{g-1}{2} \end{array}\right.$ and N is the sample size. Based on this, $\alpha_{1}^{3}$ and $\psi^{3}$ are estimated by $\max_{\alpha_{1}^{3},\psi^{3}\;}lnL$.

## 4 Results

### 4.1 Benchmark regressions

Benchmark regression results are presented in Table 2. Column (1) uses the OLS model. Columns (2)–(3) exhibit results applying the Probit and Logit models respectively. The Ordered Probit and Ordered Logit models are adopted in Columns (4)–(5) respectively. Results show that estimated coefficients of automation are significantly positive at the 1 % level in all regressions, which means that workplace automation significantly increases energy poverty. Moreover, the positive relationship between automation and energy poverty is very robust and not affected by model selection. This provides preliminary evidence for Hypothesis 2. Besides, the estimates of control variables are basically consistent with theoretical analysis and existing literature. Energy poverty is significantly negatively correlated with household income [36,71,72]. Compared with urban areas, rural areas exhibit higher incidence of energy poverty [45]. Ethnic minority groups tend to use more lower-quality solid fuels, making them more vulnerable to energy poverty [40,41]. Higher-educated family representatives have higher cognitive ability, which can help to increase household income and thus keeps them out of energy poverty [1,47]. Because those with better health status have higher human capital and migrants have stronger ability to switch jobs, they are less likely to be energy poor. In addition, the age of household representatives is also correlated with household energy poverty and the relationship is non-linear [43,44].

**Table 2:** Benchmark regressions.

Model	(1) OLS	(2) Probit	(3) Logit	(4) Oprobit	(5) Ologit
Variable	Energy_poverty	Energy_poverty	Energy_poverty	Energy_poverty	Energy_poverty
Automation	0.081*** (0.026)	0.326*** (0.118)	0.495** (0.195)	0.323*** (0.104)	0.550*** (0.181)
ln_Income	−0.043*** (0.007)	−0.252*** (0.065)	−0.492*** (0.092)	−0.161*** (0.029)	−0.287*** (0.058)
Number of houses	0.018* (0.010)	0.093* (0.050)	0.151* (0.082)	0.070* (0.038)	0.116* (0.065)
Whether owning cars	0.017 (0.018)	0.098 (0.085)	0.199 (0.141)	0.060 (0.072)	0.136 (0.127)
Social status	0.004 (0.005)	0.028 (0.022)	0.052 (0.037)	0.015 (0.018)	0.024 (0.032)
Family size	0.004 (0.006)	0.018 (0.026)	0.037 (0.044)	0.018 (0.022)	0.032 (0.038)
Number of children	0.012 (0.008)	0.040 (0.038)	0.065 (0.070)	0.047 (0.032)	0.079 (0.057)
Whether Hukou in urban areas	−0.089*** (0.019)	−0.244*** (0.090)	−0.400*** (0.150)	−0.335*** (0.077)	−0.532*** (0.134)
Whether ethnic minorities	0.095*** (0.035)	0.247* (0.149)	0.389 (0.246)	0.344*** (0.130)	0.565** (0.226)
Whether religious believers	−0.005 (0.030)	0.013 (0.134)	0.020 (0.229)	−0.008 (0.116)	−0.020 (0.201)
Whether having pension	0.008 (0.019)	0.044 (0.084)	0.059 (0.141)	0.034 (0.072)	0.044 (0.124)
Whether having medical insurance	−0.010 (0.029)	−0.252* (0.151)	−0.412 (0.256)	−0.033 (0.114)	−0.023 (0.193)
Education level	−0.009*** (0.003)	−0.032** (0.016)	−0.050* (0.027)	−0.037*** (0.013)	−0.062*** (0.023)
Health condition	−0.018** (0.008)	−0.120*** (0.038)	−0.217*** (0.064)	−0.069** (0.030)	−0.127** (0.052)
Whether migrant	−0.111*** (0.023)	−0.445*** (0.113)	−0.719*** (0.190)	−0.454*** (0.101)	−0.746*** (0.175)
Age	−0.010** (0.004)	−0.034* (0.020)	−0.053 (0.034)	−0.036** (0.016)	−0.060** (0.029)
Age_squared	0.000*** (0.000)	0.000* (0.000)	0.001* (0.000)	0.000*** (0.000)	0.001** (0.000)
Whether female	−0.019 (0.015)	−0.032 (0.069)	−0.060 (0.117)	−0.076 (0.058)	−0.135 (0.101)
Whether married	−0.007 (0.023)	0.012 (0.106)	−0.020 (0.185)	−0.042 (0.087)	−0.077 (0.153)
Whether CPC member	−0.020 (0.027)	−0.111 (0.128)	−0.179 (0.218)	−0.069 (0.110)	−0.115 (0.193)
Whether working in-system	0.007 (0.025)	0.195 (0.122)	0.331 (0.205)	0.046 (0.098)	0.088 (0.166)
Province dummies	Yes	Yes	Yes	Yes	Yes
Constant	1.032*** (0.134)	4.032*** (0.897)	7.572*** (1.341)	.	.
Observations	1773	1773	1773	1773	1773
Adjusted/Pseudo R^2^	0.270	0.185	0.186	0.163	0.159

Notes: ***, **, and * indicate significance at the levels of 1 %, 5 %, and 10 %, respectively. The values in parentheses are standard errors robust to heteroskedasticity. Yes means the corresponding variables are controlled in the regression, while No means not controlled. Adjusted R^2^ is reported for linear models and Pseudo R^2^ is reported for nonlinear models. The same for other tables below.

### 4.2 Dealing with endogeneity

Although we have comprehensively controlled factors influencing energy poverty, there may still be omitted variables in the random disturbance term, which may lead to biased estimation of automation's effect. To tackle the potential endogeneity problem, the following two-stage least squares (2SLS) statistical model is applied as equation (12), (13).

(12) $${Automation}_{i}=\beta_{0}^{1}+\beta_{1}^{1}{Routine\_Operational}_{i}+x_{i}^{\prime }\varphi^{1}+\mu_{i}^{1}$$

(13) $${Energy\_poverty}_{i}=\beta_{0}^{2}+\beta_{1}^{2}\hat{{Automation}_{i}}+x_{i}^{\prime }\varphi^{2}+\mu_{i}^{2}$$

Following Graetz and Michaels [15], we use the routine operational task-intensity measure of occupations from the *Dictionary of Occupational Titles* in 1977 by the US Department of Labor as the instrumental variable (IV) for ${Automation}_{i}$, denoted as ${Routine\_Operational}_{i}$ [73]. Because the characteristics of occupations in 1977 are related to their current features and the higher the degree of routine operational task-intensity, the easier it is to be replaced by automation [74], this instrument satisfies correlation condition. In addition, the characteristics of occupations more than 40 years ago are theoretically uncorrelated with the individual-level characteristics of current workers. Therefore, the prerequisite of exogeneity is theoretically satisfied. Model (12) is the first stage regression of 2SLS, in which ${Routine\_Operational}_{i}$ is used to estimate ${Automation}_{i}$. In model (13), the predicted values of ${Automation}_{i}$ from the first stage estimation are used to examine its effects on energy poverty.

Estimation results of the instrumental variable approach are reported in Table 3. The first stage regression results in column (1) indicate that the routine operational task-intensity in 1977 significantly enhances the degree to which the occupation is currently affected by automation at the 1 % level. In addition, the F value of this regression is 17.138, larger than the empirical criterion of 10, all of which prove that this variable is a valid instrumental variable. The estimation results of the 2SLS second stage are listed in column (2). After dealing with endogeneity, the effect of automation on energy poverty remains significantly positive at the 1 % level. This further supports Hypothesis 2 that workplace automation increases the degree of energy poverty. Moreover, to test the robustness of the instrumental variable results, we further use the Limited Information Maximum Likelihood estimation (LIML), two-step optimal Generalized Method of Moments (GMM) and iterative GMM (IGMM) for estimation. In addition, given that the dependent variable ${Energy\_poverty}_{i}$ takes values in the interval [0,1], the IV Probit model is also employed for analysis. As shown in columns (3)–(6) of Table 3, the estimates of automation are all significantly positive at the 1 % level using above different instrumental variables models. This further demonstrates that automation's impacts on energy poverty are not subject to endogeneity issues.

**Table 3:** Dealing with endogeneity: Instrumental variable regressions.

Model	(1) First Stage	(2) 2SLS Second Stage	(3) LIML Second Stage	(4) GMM Second Stage	(5) IGMM Second Stage	(6) IV Probit
Variable	Automation	Energy_poverty	Energy_poverty	Energy_poverty	Energy_poverty	Energy_poverty
Routine_ Operational	0.192*** (0.010)					
Automation		0.219*** (0.052)	0.219*** (0.052)	0.219*** (0.052)	0.219*** (0.052)	0.644*** (0.228)
Controls	Yes	Yes	Yes	Yes	Yes	Yes
Constant	0.756*** (0.110)	0.895*** (0.141)	0.895*** (0.141)	0.895*** (0.141)	0.895*** (0.141)	3.662*** (0.927)
Observations	1773	1773	1773	1773	1773	1773
Adjusted R^2^	0.353	0.277	0.277	0.277	0.277	.

### 4.3 Robustness checks

#### 4.3.1 Using different automation measures

In the conversion of automation indicators of Frey and Osborne [30] from SOC2010 to ISCO2008, when an ISCO2008 occupation corresponds to multiple SOC2010 occupations, we take the employment scale of SOC2010 occupations in the 2017 US Occupational Employment Survey (OES) as the weight and construct the weighted average indicator of automation in benchmark analysis. A natural concern is whether results of this paper depend on this specific transformation approach of the measure. With this consideration, to check whether the approach of constructing automation indicators affects our findings, we further use a variety of other indicators to check the robustness. First, when an ISCO2008 occupation corresponds to multiple SOC2010 occupations, the arithmetic average of SOC2010 occupational indicators is calculated as the ISCO2008 index, denoted as Automation_unweighted. Second, considering that both the weighted and arithmetic average indicators are susceptible to outliers, we use the median value of SOC2010 indicators to measure automation's impact on ISCO2008 occupations, denoted as Automation_median. Besides, this paper also takes a more aggressive approach for robustness test, which utilizes the maximum value of SOC2010 indicators to characterize automation's effect on ISCO2008 occupation, denoted as Automation_max.

More importantly, the following two indicators developed by other scholars, which are directly based on the ISCO2008 classification, are also applied to depict the extent to which occupations are affected by automation. The first one comes from Mihaylov and Tijden [[75], [76]]. They construct an indicator of automation's impact in the following spirit as represented in equation (14).

(14) RTI=RC+RM−NRA−NRI−NRM

where RTI indicates routine task-intensity of a specific occupation, and RC, RM, NRA, NRI and NRM stand for five task intensities: routine cognitive, routine manual, non-routine analytic, non-routine interactive and nonroutine manual, respectively. The second index is constructed by Marcolin et al. [77]. From the task perspective, using the OECD Survey of Adult Skills, they create the Routine Intensity Index (RII) as shown in equation (15) to measure the effect of automation according to occupations' characteristics in four dimensions: the frequencies with which individuals in a certain occupation may choose the sequence of the tasks involved in the work (Sequentiability), change the content of work or how it is carried out (Flexibility), plan the work activities (Plan_own), and determine their working time (Organize_own).

(15) $$RII=\omega_{seq}Sequentiability+\omega_{fle}Flexibility+\omega_{pla}Plan\_own+\omega_{org}Organize\_own$$

with the instrumental variable approach to deal with endogeneity, we further use above different automation measures to conduct robustness checks. In Table 4 and Table 5, statistical results exhibit that the effect of automation on energy poverty is significantly positive at the 1 % level no matter which indicator and which IV model is employed, further confirming the robustness of our findings. In addition, LIML, GMM and IGMM estimations are basically consistent with that using 2SLS and therefore are not demonstrated here in view of space constraints.

**Table 4:** Using different automation measures (2SLS).

Model	(1) 2SLS	(2) 2SLS	(3) 2SLS	(4) 2SLS	(5) 2SLS
Variable	Energy_poverty	Energy_poverty	Energy_poverty	Energy_poverty	Energy_poverty
Automation_unweighted	0.233*** (0.056)				
			
Automation_median		0.225*** (0.054)			
		
Automation_max			0.216***		
			(0.052)		
RTI				0.439*** (0.138)	
			
RII					0.167*** (0.040)
		
Controls	0.884*** (0.143)	0.889*** (0.142)	0.868*** (0.147)	1.297*** (0.172)	0.731*** (0.170)
Constant	1773	1773	1773	1827	1831
Adjusted R^2^	0.252	0.251	0.243	.	0.143
Observations	1773	1773	1773	1831	1827

**Table 5:** Using different automation measures (IV Probit).

Model	(1) IV Probit	(2) IV Probit	(3) IV Probit	(4) IV Probit	(5) IV Probit
Variable	Energy_poverty	Energy_poverty	Energy_poverty	Energy_poverty	Energy_poverty
Automation_unweighted	0.682*** (0.240)				
			
Automation_median		0.659*** (0.232)			
			
Automation_max			0.629*** (0.221)		
			
RTI				1.048*** (0.279)	
		
RII					0.474*** (0.147)
Controls	Yes	Yes	Yes	Yes	Yes
Constant	3.619*** (0.943)	3.637*** (0.934)	3.553*** (0.964)	3.909*** (0.862)	3.013*** (1.023)
Observations	1773	1773	1773	1827	1831

#### 4.3.2 Placebo tests

If the impact of automation on energy poverty is due to other omitted random factors, then even using the instrumental variable approach conclusions may not be credible. To test whether the estimation results of this paper are confounded by other unobservable random elements, the following placebo tests are performed. Specifically, we randomly reallocate the automation indicator in the sample and obtain a new indicator, denoted as ${Automation\_placebo}_{i}$, for each observation. Then, the new samples are used for regression to obtain estimates of ${Automation\_placebo}_{i}$’s effect on ${Energy\_poverty}_{i}$. To fully check the robustness of the results, the above process is repeated 1000 times.

**Fig. 3:**
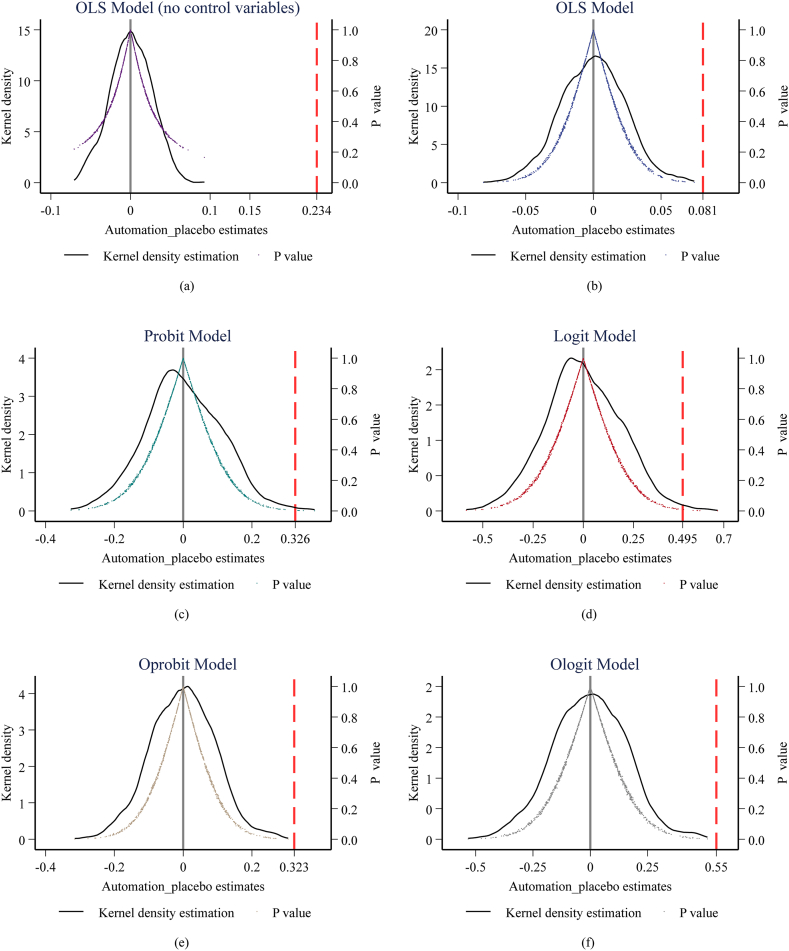
(a) Placebo test results of the OLS model without control variables, (b) placebo test results of the OLS model with control variables, (c) placebo test results of the Probit model with controls, (d) placebo test results of the Logit model with controls, (e) placebo test results of the Oprobit model with controls, (f) placebo test results of the Ologit model with controls.

In subfigures (a)–(f) of Fig. 3, the six sub figures are placebo test results respectively using OLS model without and with controls, Probit model, Logit model, Ordered Probit model, and Ordered Logit model. The black solid lines are the probability density curves of ${Automation\_placebo}_{i}$’s estimated coefficients in the 1000 new samples. The scatters are the P values corresponding to the estimates of ${Automation\_placebo}_{i}$’s parameters. The red vertical dashed lines denote the real estimates from benchmark regressions. It is clear that the regression coefficients all center around 0, basically demonstrating normal distributions, and nearly 90 % of their corresponding P values are greater than 0.1. Furthermore, all the estimated coefficients in benchmark regressions locate in the periphery of the kernel density estimation curves and are much larger than ${Automation\_placebo}_{i}$’s estimates. This suggests that the influence of automation on energy poverty cannot be attributed to omitted random factors, thus proving the robustness of our results.

#### 4.3.3 Penalized machine learning estimations

**Fig. 4:**
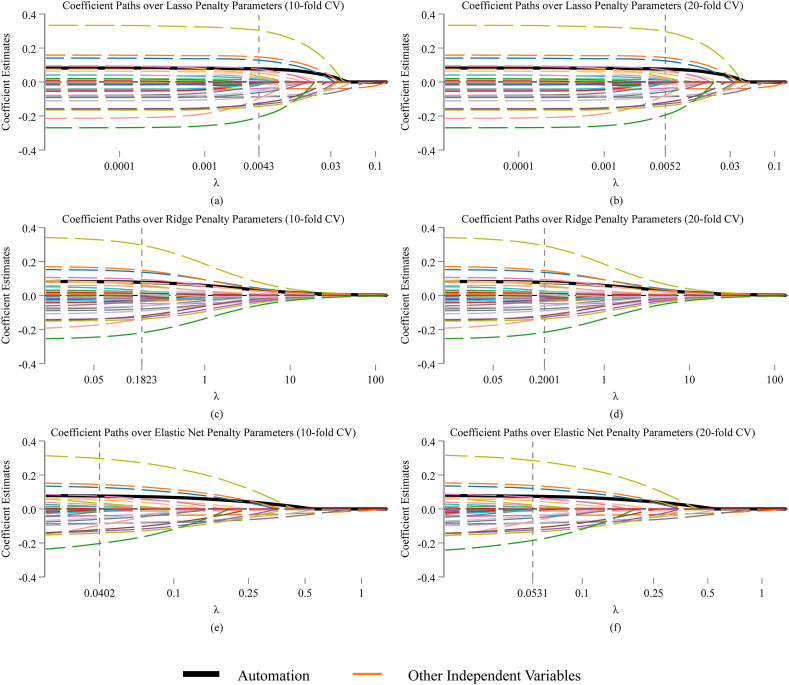
(a) Coefficients paths over Lasso penalty parameters (10-fold CV), (b) coefficients paths over Lasso penalty parameters (20-fold CV), (c) coefficients paths over Ridge penalty parameters (10-fold CV), (d) coefficients paths over Ridge penalty parameters (20-fold CV), (e) coefficients paths over Elastic Net penalty parameters (10-fold CV), (f) coefficients paths over Elastic Net penalty parameters (20-fold CV).

Since existing literature has not focused on the impact of automation on energy poverty, we further test whether automation has important and robust explanatory power for energy poverty compared with other factors that have been investigated in previous studies. First, this paper utilizes the Lasso model of machine learning for analysis. Based on 10-fold and 20-fold cross-validation, we obtain the optimal penalty parameters λ, which are 0.0043 and 0.0052 respectively. As shown in columns (1)–(2) of Table 6, under the optimal penalties, automation is among the non-zero independent variables in the both models with positive estimates. This proves that automation is a necessary element for predicting household energy poverty. Columns (3)–(6) present that findings from Ridge and Elastic Net models are consistent with that with Lasso, where automation remains a necessary indicator for predicting energy poverty in all penalized models. Furthermore, taking the penalty parameter λ as the horizontal axis, the evolution paths of independent variables' estimated coefficients in the Lasso, Ridge and Elastic Net regressions are plotted in the subfigures (a)–(f) of Fig. 4, in which automation's coefficient path is the black bold line. It is clearly depicted that the estimated coefficients of automation are all positive at optimal penalties and converge to zero until the penalties are increased to about 10 times of the optimal ***λ***. This indicates that the explanatory power of automation for energy poverty is very robust compared to other factors.

**Table 6:** Penalized machine learning estimations.

Model	(1) Lasso (10-fold CV)	(2) Lasso (20-fold CV)	(3) Ridge (10-fold CV)	(4) Ridge (20-fold CV)	(5) Elastic Net (10-fold CV)	(6) Elastic Net (20-fold CV)
Variable	Energy _poverty	Energy _poverty	Energy _poverty	Energy _poverty	Energy _poverty	Energy _poverty
Automation	0.075	0.073	0.076	0.076	0.075	0.073
Number of nonzero coefficients	34	30	49	49	35	31
Out-of-sample R^2^	0.2482	0.2484	0.2478	0.2465	0.2487	0.2488
λ	0.0043	0.0052	0.1823	0.2001	0.0402	0.0531
α					0.1	0.1
Observations	1773	1773	1773	1773	1773	1773

## 5 Analysis of different energy poverty measures

Considering the great variations in energy poverty measures in existing studies, we further construct different types of energy poverty indicators from different perspectives to examine the effects of automation.

### 5.1 Using different energy affordability thresholds of expenses

In benchmark regressions, we use the criterion of whether energy expense exceeds 10 % of total household expenditure to measure energy affordability. Although the 10 % economic threshold has been adopted in many studies (e.g., [63,64]), it is still controversial. This criterion is originally used to assess household energy affordability as a measure of energy poverty in developed countries such as Europe and the United States. Many scholars argue that the choice of this cut-off is arbitrary and possibly too low [39,78]. Especially for underdeveloped countries, where average household income is low and the share of energy expense is high, the 10 % threshold may overestimate their true level of energy poverty. To examine whether the choice of different thresholds affects the findings of this paper, referring to Churchill and Smyth [79], we further utilize 15 % and 20 % thresholds to measure energy affordability. Similarly, based on equation (1), the new energy poverty indicators are obtained, denoted as Energy_poverty_2 and Energy_poverty_3, respectively. Results in Table 7 show that automation significantly increases energy poverty at the 1 % level, regardless of whether a 10 %, 15 % or 20 % threshold is used, and no matter whether 2SLS or IV Probit model is chosen to deal with endogeneity.

**Table 7:** Using different energy affordability thresholds of expenses.

Model	(1) 2SLS	(2) IV Probit	(3) 2SLS	(4) IV Probit	(5) 2SLS	(6) IV Probit
Variable	Energy _poverty	Energy _poverty	Energy _poverty_2	Energy _poverty_2	Energy _poverty_3	Energy _poverty_3
Automation	0.219*** (0.052)	0.644*** (0.228)	0.220*** (0.051)	0.780*** (0.223)	0.243*** (0.050)	0.979*** (0.215)
Controls	Yes	Yes	Yes	Yes	Yes	Yes
Constant	0.895*** (0.141)	3.662*** (0.927)	0.805*** (0.140)	2.404*** (0.747)	0.729*** (0.143)	1.768** (0.690)
Observations	1773	1773	1773	1773	1773	1773
Adjusted R^2^	0.257	.	0.236	.	0.230	.

### 5.2 Using energy affordability thresholds in terms of income

Because people's daily expenses are scattered in many different aspects, their responses on total expenditure tend to be less accurate than income [80]. As a result, there may exist measurement errors to some extent in using thresholds of total expenditure as a criterion for energy affordability. Based on this consideration, following the energy poverty indicators of Boardman [81], Scarpellini et al. [71] and Ma et al. [35], we also adopt the standard of whether energy consumption exceeds 10 % of total income to measure energy affordability. Based on equation (1), we obtain the new energy poverty index, denoted as Energy_poverty_4. Likewise, this paper also uses 15 % and 20 % thresholds to test the robustness of the results, corresponding indicators denoted as Energy_ poverty_5 and Energy_ poverty_6, respectively. Regression results based on these measures are shown in Table 8, where workplace automation increases the severity of energy poverty, using varied energy affordability thresholds in terms of income with different instrumental variable models.

**Table 8:** Using energy affordability thresholds in terms of income.

Model	(1) 2SLS	(2) IV Probit	(3) 2SLS	(4) IV Probit	(5) 2SLS	(6) IV Probit
Variable	Energy _poverty_4	Energy _poverty_4	Energy _poverty_5	Energy _poverty_5	Energy _poverty_6	Energy _poverty_6
Automation	0.196*** (0.051)	0.486** (0.223)	0.190*** (0.049)	0.628*** (0.220)	0.201*** (0.048)	0.838*** (0.214)
Controls	Yes	Yes	Yes	Yes	Yes	Yes
Constant	1.192*** (0.147)	6.684*** (0.770)	1.168*** (0.140)	5.547*** (0.748)	1.099*** (0.132)	4.833*** (0.738)
Observations	1773	1773	1773	1773	1773	1773
Adjusted R^2^	0.318	.	0.304	.	0.284	.

### 5.3 Energy poverty measures considering energy subsidy

In benchmark analysis, we use the share of total household energy expenditure to define energy affordability. Actually, some Chinese households receive energy subsidies provided by the government, such as low-income households below the poverty line and those that meet the government's “coal-to-electricity” transition policy. Therefore, the energy poverty indicator based on total energy expenditure may overestimate the real energy poverty level and fail to objectively measure the impact of workplace automation. To test the robustness of the conclusions, we deduct governmental energy subsidies from the total energy expense and construct energy poverty indicators using net energy expenditure with different thresholds. Energy_poverty_7, Energy_poverty_8 and Energy_poverty_9 denote energy poverty indicators with 10 %, 15 % and 20 % thresholds respectively, for which the affordability is characterized by the percentage of net energy expenses in household expenditures excluding energy subsidies. Results in Table 9 show that no matter which model and threshold are chosen, after subtracting energy subsidies, the estimated coefficients of automation are all significantly positive at the 1 % level, providing further evidence that automation increases energy poverty.

**Table 9:** Energy poverty measures considering energy subsidy.

Model	(1) 2SLS	(2) IV Probit	(3) 2SLS	(4) IV Probit	(5) 2SLS	(6) IV Probit
Variable	Energy _poverty_7	Energy _poverty_7	Energy _poverty_8	Energy _poverty_8	Energy _poverty_9	Energy _poverty_9
Automation	0.214*** (0.053)	0.637*** (0.227)	0.211*** (0.052)	0.736*** (0.224)	0.238*** (0.050)	0.946*** (0.216)
Controls	Yes	Yes	Yes	Yes	Yes	Yes
Constant	0.903*** (0.141)	3.688*** (0.931)	0.834*** (0.141)	2.539*** (0.752)	0.740*** (0.144)	1.880*** (0.698)
Observations	1773	1773	1773	1773	1773	1773
Adjusted R^2^	0.241	.	0.219	.	0.214	.

### 5.4 Using relative energy poverty measures

The energy poverty indicators used above are all in absolute sense. However, poverty is a relative concept. When talking about poverty, we should not only consider individual characteristics, but also embed them in specific socioeconomic contexts and compare them with their counterparts. Therefore, relative indicators should also be considered to measure energy poverty. In view of this, referring to Thema and Vondung [82], Simshauser [83], and Chen and Feng [84], a relative energy poverty indicator is also constructed in this study. This indicator measures relative energy poverty in the following two dimensions. First, identify relatively poor households. Specifically, if the household income before or after energy costs is less than 60 % of the median income of all households, the household is considered as relatively poor from the perspective of income. The first indicator is constructed in the way shown in equation (16). In addition, because of the energy-sharing effect among family members living together [85], energy cost of a family does not increase to the same extent as the family size increases. Consequently, family size should also be taken into account and equivalent income and equivalent energy expenses are constructed following OECD [86] based on equations (17), (18). Furthermore, we use the equivalent indicators to identify relatively poor families, the corresponding dummy variable denoted as equivalent_income_poor in equation (19).

(16) $$income\_poor=\left\{ \begin{array}{c} 1\;if\;income\;before\;or\;after\;energy\;cost<60\%\;of\;the\;median\;income\\ 0\;if\;income\;before\;or\;after\;energy\;cost\geq 60\%\;of\;the\;median\;income \end{array}\right.$$

(17) $$equivalent\;income=income/\sqrt{family\;size}$$

(18) $$equivalent\;energy\;expenses=energy\;expense/\sqrt{family\;size}$$

(19) $$equivalent\_income\_poor=\left\{ \begin{array}{c} 1\;if\;equivalent\;income\;before\;or\;after\;equivalent\;energy\;cost<60\%\;of\;the\;median\;equivalent\;income\\ 0\;if\;equivalent\;income\;before\;or\;after\;equivalent\;energy\;cost\geq 60\%\;of\;the\;median\;equivalent\;income \end{array}\right.$$

Second, identify relatively low energy expense families. If a household spends less on energy, it may not be able to meet basic energy needs. Specifically, a household is classified in the category of relatively low energy expense if its energy expenditure is less than half that of the median household [82]. The second indicator is defined as equation (20). Moreover, similarly, we also build this indicator using net household energy expense, the corresponding dummy variable denoted as low_net_energy_expense, as shown in equation (21).

(20) $$low\_energy\_expense=\left\{ \begin{array}{c} 1\;if\;energy\;expense<50\%\;of\;the\;median\;energy\;expense\\ 0\;if\;energy\;expense\geq 50\%\;of\;the\;median\;energy\;expense \end{array}\right.$$

(21) $$low\_net\_energy\_expense=\left\{ \begin{array}{c} 1\;if\;net\;energy\;expense<50\%\;of\;the\;median\;net\;energy\;expense\\ 0\;if\;net\;energy\;expense\geq 50\%\;of\;the\;median\;net\;energy\;expense \end{array}\right.$$

Based on the above two aspects, the relative energy poverty indicator is constructed as follows. If the household is neither relatively poor in terms of income nor relatively low in energy expense, the relative energy poverty indicator is 0. If the household is not relatively poor in terms of income but relatively low in energy expense, the relative energy poverty indicator is 1. If the household is relatively poor in terms of income but not relatively low in energy expense, the relative energy poverty indicator is 2. If the household is both relatively poor in terms of income and relatively low in energy expense, the relative energy poverty indicator is 3. Energy_poverty_10 is the relative energy poverty indicator constructed based on income_poor and low_energy_expense. Energy_poverty_11 is the indicator using income_poor and low_net_energy_expense. Energy_poverty_12 is the indicator built with equivalent_income_poor and low_energy_expense. Energy_poverty_13 is the indicator based on equivalent_income_poor and low_net_energy_expense.

**Fig. 5:**
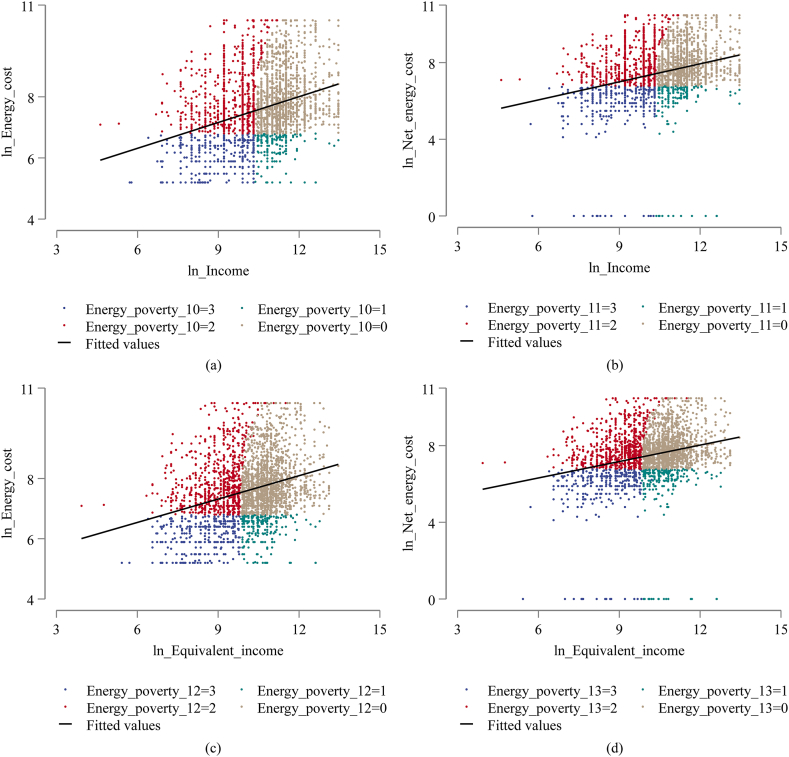
Distribution of households in different relative energy poverty categories for (a) ln_Income and ln_Energy_cost, (b) ln_Income and ln_Net_energy_cost, (c) ln_Equivalent_income and ln_Energy_cost, (d) ln_Equivalent_income and ln_Net_Energy_cost.

Scatter plots of the relationship between household income and energy expense are illustrated in subfigures (a)–(d) of Fig. 5 display the distribution of households with different levels of energy poverty under the above four relative energy poverty measures. The black fitted line sloping upwards to the right in the figures reflects a positive relationship between household income and energy consumption. In addition, most of the households are at a medium level of energy poverty, taking a value of 1 or 2 for the relative energy poverty indicator. Regression results using the above four relative energy poverty indicators are illustrated in Table 10. The estimated coefficients of automation are all significantly positive at the 1 % level, further corroborating that automation increases energy poverty. This means that with relative energy poverty measures, the greater people's work is affected by automation, the more prominent the energy poverty problem they face relative to their counterparts.

**Table 10:** Using relative energy poverty measures.

Model	(1) 2SLS	(2) 2SLS	(3) 2SLS	(4) 2SLS
Variable	Energy _poverty_10	Energy _poverty_11	Energy _poverty_12	Energy _poverty_13
Automation	0.407*** (0.130)	0.417*** (0.129)	0.423*** (0.127)	0.434*** (0.126)
Controls	Yes	Yes	Yes	Yes
Constant	3.742*** (0.435)	3.729*** (0.436)	3.535*** (0.438)	3.522*** (0.439)
Observations	1773	1773	1773	1773
Adjusted R^2^	0.545	0.543	0.540	0.537

## 6 Mechanism analysis

Preceding sections of this paper have confirmed Hypothesis 2 that automation increases energy poverty. So, through what mechanisms does workplace automation exert its impact on energy poverty? Referring to Alesina et al. [[87], [88], [89]], the following mechanism model based on the instrumental variable approach, represented in equations (22), (23), is constructed:

(22) $${Mediator}_{i}=\gamma_{0}^{1}+\gamma_{1}^{1}\hat{{Automation}_{i}}+x_{i}^{\prime }\varphi^{1}+\epsilon_{i}^{1}$$

(23) $${Energy\_poverty}_{i}=\gamma_{0}^{2}+\gamma_{1}^{2}\hat{{Automation}_{i}}+\gamma_{2}^{2}{Mediator}_{i}+x_{i}^{\prime }\varphi^{2}+\epsilon_{i}^{2}$$

${Mediator}_{i}$ is the mediating variable. $\hat{{Automation}_{i}}$ is the predicted value of automation in the first stage regression of 2SLS in equation (12). Models (22)–(23) use $\hat{{Automation}_{i}}$ for the second stage regression of 2SLS. If both $\gamma_{1}^{1}$ in model (22) and $\gamma_{2}^{2}$ in model (23) are estimated to be significant, ${Mediator}_{i}$ would be proven to mediate automation's impact on energy poverty.

### 6.1 Income mechanism

It has been documented that automation's replacement effect on workers as well as the resulting mismatches in the labor market bring down people's income [26,27,[30], [31], [32],^90^]. Meanwhile, economic income is one of the most direct and important factors affecting energy poverty. Low-income households are more likely to fall into energy poverty [35,36]. Based on this, we propose the conjecture that households affected by automation to a larger extent may have lower income and consequently be more vulnerable to energy poverty. Columns (1)–(2) in Table 11 are estimated results of 2SLS and IV Probit models for the impact of automation on energy poverty when income is not controlled for, showing that automation significantly increases energy poverty. Results in column (3) of Table 11 illustrate that automation significantly reduces household income. Meanwhile, in columns (4)–(5), income has a significant negative effect on energy poverty, implying that households with higher income are less likely to be energy poor. In addition, when household income is included in the regressions, the estimated coefficients of automation in columns (4)–(5) drop compared with that in columns (1)–(2). This proves that income mediates automation's effect on energy poverty. Therefore, automation exposes households to higher levels of energy poverty by lowering income.

**Table 11:** Income mechanism analysis.

Model	(1) 2SLS	(2) IV Probit	(3) 2SLS	(4) 2SLS	(5) IV Probit
Variable	Energy_poverty	Energy_poverty	ln_Income	Energy_poverty	Energy_poverty
Automation	0.251*** (0.052)	0.789*** (0.216)	−0.782*** (0.231)	0.219*** (0.052)	0.644*** (0.228)
ln_Income				−0.041*** (0.007)	−0.243*** (0.064)
		
Other Controls	Yes	Yes	Yes	Yes	Yes
Constant	0.520*** (0.122)	1.349** (0.555)	9.202*** (0.578)	0.895*** (0.141)	3.662*** (0.927)
Observations	1773	1773	1773	1773	1773
Adjusted R^2^	0.231	.	0.393	0.257	.

Notes: Other controls in this table refer to control variables excluding the logarithm of family income.

### 6.2 Work-related social capital mechanism

The benefits that work brings to people include not only economic income, but also social capital. Social capital facilitates resource and information sharing, providing valuable social support. This helps mitigate the negative impact of adverse economic conditions on individuals, reducing the likelihood of falling into energy poverty, especially when faced with replacement by new technologies [19,20,91]. Moreover, social capital can be converted into both material and human capital, further strengthening the ability to resist the risks of energy poverty [1,28,92]. So, does automation also reduce work-related social capital and consequently increase the level of energy poverty? To test this hypothesis, from the question in CGSS, “How often do you use your current job to benefit your friends and relatively?”, we construct the mediating variable “work-related social capital”. Answers to this question, based on the 5-Point Likert Scale, classify the frequency from 1 to 5 as never, seldom, sometimes, often and frequently. Obviously, higher frequency means more social capital gained from work. Estimation results in column (3) of Table 12 show that workplace automation has significantly negative effect on work-related social capital. Also, it is indicated in columns (4)–(5) that work-related social capital can help to decrease energy poverty, which is consistent with findings in above existing literature. Moreover, after controlling for work-related social capital, the estimated coefficients of automation decline, implying that work-related social capital mediates automation's impact on energy poverty.

**Table 12:** Work-related social capital mechanism analysis.

Model	(1) 2SLS	(2) IV Probit	(3) 2SLS	(4) 2SLS	(5) IV Probit
Variable	Energy_poverty	Energy_poverty	Work-related social capital	Energy_poverty	Energy_poverty
Automation	0.219*** (0.052)	0.644*** (0.228)	−0.969*** (0.149)	0.174*** (0.054)	0.511** (0.239)
Work-related social capital				−0.048*** (0.010)	−0.161*** (0.047)
		
Controls	Yes	Yes	Yes	Yes	Yes
Constant	0.895*** (0.141)	3.662*** (0.927)	1.791*** (0.388)	0.981*** (0.141)	3.871*** (0.925)
Observations	1773	1773	1770	1770	1770
Adjusted R^2^	0.257	.	0.144	0.274	.

## 7 Heterogeneity analysis

Previous results reveal that workplace automation poses a new challenge of achieving SDG 7 in the context of technological revolution. So, how can we mitigate automation's adverse effects on energy consumption while taking advantage of this important technological progress? Based on heterogeneity analysis, this paper further explores potential ways to weaken the negative impact of automation from multiple perspectives. The following moderating effect model based on the instrumental variable approach is employed for analysis as represented in equations (24), (25), (26):

(24) $${Automation}_{i}=\delta_{0}^{1}+\delta_{1}^{1}{Routine\_Operational}_{i}+\delta_{2}^{1}{Routine\_Operational}_{i}*{Moderator}_{i}+x_{i}^{\prime }\zeta^{1}+\nu_{i}^{1}$$

(25) $${Automation}_{i}*{Moderator}_{i}=\delta_{0}^{2}+\delta_{1}^{2}{Routine\_Operational}_{i}+\delta_{2}^{2}{Routine\_Operational}_{i}*{Moderator}_{i}+x_{i}^{\prime }\zeta^{2}+\nu_{i}^{2}$$

(26) $${Energy\_poverty}_{i}=\delta_{0}^{3}+\delta_{1}^{3}\hat{{Automation}_{i}}+\delta_{2}^{3}\hat{{Automation}_{i}*{Moderator}_{i}}+x_{i}^{\prime }\zeta^{3}+\nu_{i}^{3}$$

${Moderator}_{i}$ represents the moderating variable. Here we particularly focus on $\delta_{2}^{3}$. If it is significantly larger than 0, it would be proven that the moderator enhances automation's impact on energy poverty. Conversely, if $\delta_{2}^{3}$ is significantly negative, it would mean that the moderator alleviates automation's shock.

### 7.1 Heterogeneities of urban-rural background, human capital and labor protection

First of all, considering that there are significant urban-rural disparities in China's economic and social development, this paper examines the heterogeneity in terms of urban-rural background. As presented in Table 13 column (1), the estimated coefficient of the interaction term between automation and the dummy variable of whether in urban areas is significantly negative, indicating that automation has less negative effects on urban households. This may be due to the fact that energy infrastructure is better in urban areas, so urban households have more access to modern high-quality energy services, which makes them less susceptible to energy poverty. As a result, workplace automation's impact on urban households is less pronounced. Second, with respect to human capital, results in columns (2)–(3) of Table 13 show that higher educated workers and migrants are affected by automation shock to a much less extent. This may be due to the fact that highly educated workers have more human capital, and thus it is easier for them to acquire new skills and find new jobs. Migrants are more likely to change jobs by switching locations when their work is substituted. This also means that increasing human capital investment and promoting free movement of labor can mitigate the adverse effect of automation on energy poverty. Third, we also discuss the role of labor protection. In columns (4)–(6) of Table 13, for those with labor contracts, trade union members, and in-system workers, automation has a much smaller impact on energy poverty. This suggests that providing better labor protection for workers also contributes to weakening the side impact of automation technology. To check the robustness of above findings, we also use the IV Probit model for moderating effect regressions and Table A1 exhibits that the same conclusions can be obtained.

**Table 13:** Heterogeneities of urban-rural background, human capital and labor protection.

Model	(1) 2SLS	(2) 2SLS	(3) 2SLS	(4) 2SLS	(5) 2SLS	(6) 2SLS
Variable	Energy _poverty	Energy _poverty	Energy _poverty	Energy _poverty	Energy _poverty	Energy _poverty
Automation	0.350*** (0.065)	0.554*** (0.095)	0.274*** (0.056)	0.532*** (0.135)	0.265*** (0.058)	0.283*** (0.061)
Whether Hukou in urban areas *Automation	−0.447*** (0.097)					
				
Education level *Automation		−0.063*** (0.013)				
				
				
Whether migrant *Automation			−0.439*** (0.142)			
				
				
Whether having labor contract *Automation				−0.501*** (0.153)		
				
				
Whether trade union member *Automation					−0.287*** (0.110)	
				
				
Whether working in-system *Automation						−0.332*** (0.104)
				
				
Moderator	Yes	Yes	Yes	Yes	Yes	Yes
Other Controls	Yes	Yes	Yes	Yes	Yes	Yes
Constant	0.819*** (0.143)	0.677*** (0.151)	0.854*** (0.141)	0.679*** (0.175)	0.870*** (0.142)	0.855*** (0.142)
Observations	1773	1773	1773	1764	1754	1773
Adjusted R^2^	0.254	0.261	0.257	0.242	0.260	0.255

Notes: Other controls in this table refer to control variables excluding the corresponding moderator.

### 7.2 Heterogeneities of energy supply

Above analysis has demonstrated the variations of automation's effect on energy poverty in households with different socioeconomic backgrounds on the demand side. So, from the perspective of supply side, is better energy supply able to weaken the adverse impact of automation? To answer this question, five aspects measuring the quality of energy supply, including price reasonability, stability, security, accessibility and convenience in use, are discussed in this paper. Each aspect of the energy supply quality is scored by respondents on a scale of 1–10, with 10 being the best. Estimated coefficients of the interactions in columns (1)–(5) of Table 14 are all significantly negative, indicating that improving price reasonability, stability, security, accessibility and convenience in use of energy helps to alleviate the adverse effect of automation on energy poverty. This implies that better energy supply is conducive to attenuating the effect of automation on households' energy consumption. Likewise, we also use the IV Probit model for robustness checks, shown in Table A2, and obtain the same conclusions, which suggest that the higher the quality of energy supply, the less the adverse impact of automation on household energy use.

**Table 14:** Heterogeneities of energy supply.

Model	(1) 2SLS	(2) 2SLS	(3) 2SLS	(4) 2SLS	(5) 2SLS
Variable	Energy_poverty	Energy_poverty	Energy_poverty	Energy_poverty	Energy_poverty
Automation	0.441*** (0.105)	0.520*** (0.108)	0.489*** (0.112)	0.412*** (0.078)	0.383*** (0.077)
Price_reasonability *Automation	−0.064** (0.026)				
			
Price_reasonability	0.072*** (0.018)				
			
Energy_stability *Automation		−0.075*** (0.024)			
			
Energy_stability		0.086*** (0.016)			
			
Energy_security *Automation			−0.069*** (0.026)		
			
Energy_security			0.084*** (0.018)		
			
Energy_access_conve *Automation				−0.076*** (0.023)	
			
Energy_access_conve				0.076*** (0.016)	
			
Energy_using_conve *Automation					−0.066*** (0.024)
			
Energy_using_conve					0.070*** (0.016)
			
Controls	Yes	Yes	Yes	Yes	Yes
Constant	0.674*** (0.151)	0.599*** (0.151)	0.620*** (0.152)	0.727*** (0.148)	0.749*** (0.147)
Observations	1771	1773	1773	1738	1738
Adjusted R^2^	0.271	0.281	0.284	0.270	0.271

## 8 Discussion

Automation represents a global trend, driven by the substantial economic benefits it offers. Recognizing these advantages, major countries worldwide have formulated strategies to actively promote the development of automation technology. For instance, the United States launched the National Robotics Initiative (NRI) in 2013, Japan proposed the New Robot Strategy in 2015, the European Union released Horizon Europe in 2021, and China unveiled the “14th Five-Year Plan” for Robot Industry Development [93] in 2021. These initiatives significantly contribute to the rapid advancement of automation technologies, including artificial intelligence. However, as mentioned by Acemoglu and Restrepo [94], the wrong kind of automation may lead to negative consequences. From an energy demand perspective, this paper's findings demonstrate that automation significantly increases household energy poverty, mainly due to diminished incomes and a decline in work-related social capital. The adverse effects of automation on the labor market primarily manifest through the substitution of workers, a phenomenon observed across numerous countries and regions. Frey and Osborne's [30] study, evaluating the susceptibility of 700 occupations to automation, estimates that up to 47 % of U.S. workers are engaged in easily automated jobs, with only one-third in lower-risk occupations. This substitution effect is also observed in South Africa [95], Latin America [96], and Europe [97]. Taking European employment as an example, it is estimated that within a decade, 47 % of jobs will undergo automation, with 35 % being fully automated [98]. Therefore, considering the widespread impact of automation on the global labor market, this paper's findings hold significant practical implications globally. This necessitates policymakers to regulate and guide the direction of technological progress while protecting workers' interests.

Furthermore, energy poverty is widely prevalent across the world. Established studies have explored the factors affecting the energy poverty from various aspects, including economic income, household characteristics, human capital characteristics, policies and legislations, and financial programs [35,41,52]. Simultaneously, relevant institutions have taken measures to address energy poverty. For instance, in 2021, the European Union issued its first Recommendation on energy poverty (EU/2020/1563) [99], and the Chinese government proposed the “Promoting Alternatives to Electricity” and the “coal to electricity” plan [100]. However, these studies and approaches relatively overlook the impact of technological changes. This study's findings reveal that automation exacerbates energy poverty, and this problem is likely to worsen as automation becomes more widespread. Therefore, the analysis of the relationship between automation and energy poverty extends the existing literature on factors influencing energy poverty, providing valuable insights for formulating relevant policies.

This paper systematically investigates the impact of workplace automation on energy poverty from a demand-side perspective. The results reveal a robust finding that workplace automation significantly increases household energy poverty, with its mechanism involving reductions in income and work-related social capital. Heterogeneity analyses demonstrate a milder impact on urban households, while higher-educated workers and migrants are less affected. Labor protection measures, such as contracts and trade union membership, weaken automation's effect. Strategies like increased human capital investment, worker mobility promotion, and enhanced labor protection collectively mitigate automation's impact. Furthermore, improvements in energy price reasonability, stability, security, accessibility, and convenience alleviate the negative consequences of workplace automation on household energy use. Compared to the established literature, the contributions of this paper are reflected in the following two aspects. This paper identifies a new challenge to SDG 7, considering technological disruption's impact on energy poverty. While existing literature extensively addresses factors like economic income [36,72,101], family demographics, and social characteristics [37,40,45,46,92], along with regional influences [50,51,100], it relatively overlooks the impact of new technological changes on energy poverty. Against the backdrop of the current fourth technological revolution, this study fills this gap by explaining the growing significance of the new technological landscape in influencing energy poverty. Consequently, it offers insights into the complexities and challenges associated with achieving SDG 7. Second, this study examines the impact of digital disruption on the energy market from a demand perspective. Prior research has predominantly focused on the supply-side effects of automation on the energy market. Studies indicate that automation's positive influence on energy efficiency and structure is beneficial in alleviating energy poverty. For instance, automation technologies can optimize energy supply through intelligent algorithms and automated devices, reducing waste and enhancing energy supply efficiency [7,8]. Moreover, the application of automation in various industries reduces the demand for traditional energy sources, fostering energy transition [9]. However, the indirect impact of automation on energy poverty remains unexplored in previous studies, making this paper a valuable exploration in this area.

## 9 Conclusions and policy implications

This paper systematically examines how workplace automation impacts energy poverty from a demand-side perspective and investigates the impact mechanisms. On this basis, we demonstrate the heterogeneity of this impact across different groups. The main findings and policy implications of our study are as follows: First, workplace automation significantly increases the level of household energy poverty. This finding is robust when using the instrumental variable approach to tackle endogeneity, and employing different automation and energy poverty measures, placebo tests, and machine learning methods for robustness checks. Therefore, policymakers should attach great importance to the impact of workplace automation on energy poverty. With the large-scale application of automation, more targeted social security policies should be developed to improve household energy affordability. At the same time, it is necessary to promote the accessibility of modern clean energy.

Second, automation's impact mechanism is that it reduces people's income and work-related social capital, thus exposing households to a higher risk of energy poverty. To address this, enhancing the education and training system is crucial. This involves empowering individuals to refine adaptable skills, promoting effective collaboration with automation, and thus decreasing the risk of job displacement. This proactive approach contributes to mitigating the impact of automation on income and social capital, ultimately reducing the risk of energy poverty. Third, its negative consequences are more prominent for rural households, less educated people, non-migrants, those without labor contracts, non trade-union members, and out-of-system workers. Therefore, more attention should be paid to vulnerable groups who face a higher risk of energy poverty in the technological disruptions, such as rural households, lower-educated workers, and those lacking labor protection. Governments and public agencies can consider providing more energy subsidies for them. Besides, improving the price reasonability, stability, security, accessibility and convenience in use of energy also helps mitigate the negative consequences of workplace automation on household energy use. This means more effort should be devoted to enhancing the quality of energy infrastructure in the new technological transformation. Additionally, this necessitates a multifaceted approach that not only ensures affordability and stability in energy pricing but also fortifies security measures, enhances accessibility to diverse energy sources, and optimizes user-friendly features.

## Ethical approval

Not applicable.

## Funding

This work was supported by National Natural Science Foundation of China [grant number 62203272], Shandong Province Natural Science Foundation [grant number ZR202111090067], and National Social Science Fund of China [grant number 23CJL007].

## Data availability statement

The data that support the findings of this study are available from the Chinese General Social Survey (CGSS, http://cgss.ruc.edu.cn/English/Home.htm (accessed on 16 November 2023)). Restrictions apply to the availability of these data, which were used under license for this study. Data are also available from the authors with the permission of the CGSS.

## CRediT authorship contribution statement

**Xiaoru Niu:** Conceptualization, Formal analysis, Funding acquisition, Methodology, Project administration, Resources, Writing – review & editing. **Chao Li:** Data curation, Formal analysis, Funding acquisition, Investigation, Methodology, Writing – review & editing. **Xiang Li:** Data curation, Formal analysis, Methodology, Software, Writing – review & editing. **Yuhan Zhang:** Investigation, Methodology, Resources, Software, Validation, Visualization, Writing – original draft, Writing – review & editing.

## Declaration of competing interest

The authors declare that they have no known competing financial interests or personal relationships that could have appeared to influence the work reported in this paper.

## Figures and Tables

**Table A1:** Heterogeneities of urban-rural background, human capital and labor protection (IV Probit).

Model	(1) IV Probit	(2) IV Probit	(3) IV Probit	(4) IV Probit	(5) IV Probit	(6) IV Probit
Variable	Energy _poverty	Energy _poverty	Energy _poverty	Energy _poverty	Energy _poverty	Energy _poverty
Automation	1.124*** (0.263)	2.035*** (0.406)	0.871*** (0.238)	1.893*** (0.521)	0.817*** (0.250)	0.834*** (0.257)
Whether Hukou in urban areas *Automation	−1.560*** (0.435)					
				
Education level *Automation		−0.258*** (0.059)				
				
				
Whether migrant *Automation			−1.832*** (0.659)			
				
				
Whether having labor contract *Automation				−1.954*** (0.601)		
				
				
Whether trade union member *Automation					−0.899* (0.522)	
				
				
Whether working in-system *Automation						−0.935** (0.477)
				
				
Moderator	Yes	Yes	Yes	Yes	Yes	Yes
Controls	Yes	Yes	Yes	Yes	Yes	Yes
Constant	3.320*** (0.912)	2.759*** (0.930)	3.479*** (0.912)	2.671*** (1.010)	3.588*** (0.929)	3.539*** (0.924)
Observations	1773	1773	1773	1764	1754	1773

**Table A2:** Heterogeneities of energy supply (IV Probit).

Model	(1) IV Probit	(2) IV Probit	(3) IV Probit	(4) IV Probit	(5) IV Probit
Variable	Energy_poverty	Energy_poverty	Energy_poverty	Energy_poverty	Energy_poverty
Automation	2.263*** (0.505)	2.959*** (0.567)	2.851*** (0.596)	1.884*** (0.382)	1.774*** (0.380)
Price_reasonability *Automation	−0.482*** (0.134)				
			
Price_reasonability	0.493*** (0.095)				
			
Energy_stability *Automation		−0.595*** (0.136)			
			
Energy_stability		0.597*** (0.097)			
			
Energy_security *Automation			−0.582*** (0.149)		
			
Energy_security			0.605*** (0.107)		
			
Energy_access_conve *Automation				−0.511*** (0.128)	
			
Energy_access_conve				0.493*** (0.093)	
			
Energy_using_conve *Automation					−0.475*** (0.130)
			
Energy_using_conve					0.471*** (0.094)
			
Controls	Yes	Yes	Yes	Yes	Yes
Constant	2.142** (1.008)	1.609 (1.000)	1.714* (1.014)	2.568** (1.006)	2.679*** (1.009)
